# Expression and Contributions of TRPM7 and KCa2.3/SK3 Channels to the Increased Migration and Invasion of Microglia in Anti-Inflammatory Activation States

**DOI:** 10.1371/journal.pone.0106087

**Published:** 2014-08-22

**Authors:** Tamjeed Siddiqui, Starlee Lively, Roger Ferreira, Raymond Wong, Lyanne C. Schlichter

**Affiliations:** 1 Toronto Western Research Institute, Genes and Development Division, University Health Network, Toronto, Ontario, Canada; 2 Department of Physiology, University of Toronto, Toronto, Ontario, Canada; Indiana School of Medicine, United States of America

## Abstract

Microglia rapidly respond to CNS injury and disease and can assume a spectrum of activation states. While changes in gene expression and production of inflammatory mediators have been extensively described after classical (LPS-induced) and alternative (IL4-induced) microglial activation, less is known about acquired de-activation in response to IL10. It is important to understand how microglial activation states affect their migration and invasion; crucial functions after injury and in the developing CNS. We reported that LPS-treated rat microglia migrate very poorly, while IL4-treated cells migrate and invade much better. Having discovered that the lamellum of migrating microglia contains a large ring of podosomes – microscopic structures that are thought to mediate adhesion, migration and invasion – we hypothesized that IL4 and IL10 would differentially affect podosome expression, gene induction, migration and invasion. Further, based on the enrichment of the KCa2.3/SK3 Ca^2+^-activated potassium channel in microglial podosomes, we predicted that it regulates migration and invasion. We found both similarities and differences in gene induction by IL4 and IL10 and, while both cytokines increased migration and invasion, only IL10 affected podosome expression. KCa2.3 currents were recorded in microglia under all three activation conditions and *KCNN3* (KCa2.3) expression was similar. Surprisingly then, of three KCa2.3 inhibitors (apamin, tamapin, NS8593), only NS8593 abrogated the increased migration and invasion of IL4 and IL10-treated microglia (and invasion of unstimulated microglia). This discrepancy was explained by the observed block of TRPM7 currents in microglia by NS8593, which occurred under all three activation conditions. A similar inhibition of both migration and invasion was seen with a TRPM7 inhibitor (AA-861) that does not block KCa2.3 channels. Thus, we conclude that TRPM7 (not KCa2.3) contributes to the enhanced ability of microglia to migrate and invade when in anti-inflammatory states. This will be an important consideration in developing TRPM7 inhibitors for treating CNS injury.

## Introduction

Microglial cells become activated within the CNS after acute injury and with disease, but it is increasingly clear that they exist in a spectrum of activation states and are not simply pro- or anti-inflammatory [1], [2], [3]. Mechanistic, *in vitro* studies generally exploit several stimuli to evoke discrete activation states. ‘Classical’ activation increases pro-inflammatory mediators that can exacerbate injury, and this state is commonly evoked by lipopolysaccharide (LPS) *in vitro* [1], [4]. ‘Alternative’ activation is typically evoked by interleukin (IL)-4, ‘acquired deactivation’ by IL10, and both states are thought to help resolve acute inflammation by decreasing pro-inflammatory mediators (e.g., cytokines, reactive oxygen and nitrogen species) (reviewed in [1], [5], [6], [7]). While IL4- and IL10-treatment can similarly affect some microglial responses, there is evidence for differential effects; e.g., in cytokine and chemokine secretion [8], [9] and phagocytosis [10], [11]. Moreover, a recent comparative study of murine microglia showed that IL10 evoked gene expression changes more closely resembling LPS- than IL4-treated cells [12]. Thus, it is important to compare the effects of IL4 and IL10 on specific microglial functions.

Among the earliest microglial responses to CNS injury is their migration to the damage site and contribution to remodeling the extracellular matrix (ECM), but surprisingly little is known about whether microglial activation states affect these processes. Previously, we found that LPS treatment greatly reduced microglial migration, while IL4 treatment increased migration and altered the panel of enzymes used for ECM degradation [13]. There is apparently no comparable information concerning IL10-treated microglia. Thus, one goal was to determine whether IL10 decreases migration and invasion (like LPS) or increases these important functions, like IL4. Migration and invasion of some cells corresponds with induction of tiny, protrusive, F-actin-rich structures (podosomes) that can affect both adhesion and ECM degradation [14], [15]. We recently discovered that the lamellum of unstimulated migratory microglia contains hundreds of podosomes arranged in a large ring structure [16] but it is not known if podosome expression is dependent on the microglial activation state. We also found that Ca^2+^ entry through CRAC/Orai1 channels regulates podosome expression, migration and invasion, and that the podosomes are highly enriched in the Ca^2+^-dependent KCa2.3/SK3 channel [17]. Both KCa2.3 and the Ca^2+^-permeable TRPM7 channel have been implicated in migration of several cell types (see Discussion). Because microglial invasion was reduced by the KCa2.3 inhibitor, NS8593; we speculated that KCa2.3 regulates this process by controlling podosome expression [17]. However, a surprising recent discovery is that NS8593 also blocks cloned TRPM7 channels [18]. Rat microglia have robust expression of TRPM7 transcripts and currents [19], [20], which raises the possibility that podosome expression, migration and invasion are regulated by TRPM7, rather than (or in addition to) KCa2.3.

In this study, we compared how microglial activation by IL4 or IL10 affects gene expression (28 genes), as well as migration, invasion, podosome expression, and the expression and contributions of KCa3.2 and TRPM7 channels to migration and invasion.

## Materials and Methods

### Cell cultures

#### Ethics statement

Rats were handled and sacrificed using standard operating procedures that were approved by the University Health Network Animal Care Committee (Permit number: 914.18), in accordance with guidelines from the Canadian Council on Animal Care.

Primary microglia were isolated from 1 to 2 day-old Sprague-Dawley rat pups (Charles River, St.-Constant, PQ, Canada). Neonatal microglia were used because they are naturally migratory in the healthy CNS [5]. The isolation protocol routinely used in our lab produces ≥99% pure microglia that are not activated, as judged by numerous molecular markers ([13], [21], [23]; present study). Briefly, after the meninges were peeled off, the whole brain was minced, centrifuged (300×g, 10 min) and re-suspended in Minimal Essential Medium (MEM; Invitrogen, Carlsbad, CA) supplemented with 10% fetal bovine serum (FBS; Wisent, St-Bruno, PQ, Canada), and 0.05 mg/ml gentamycin (Invitrogen). Mixed cell suspensions were seeded and cultured in tissue culture flasks for 48 hr at 37°C and 5% CO_2_. The cells were washed, and then mixed astrocyte-microglia cultures were cultured for a further 4 to 5 days in MEM with 10% FBS. The flasks were shaken (2–4 hr, 65 rpm, 37°C, 5% CO_2_) to dislodge microglia from adherent astrocytes, and the suspension was centrifuged again (300×g, 10 min) and re-suspended in MEM with 2% FBS. Microglia were plated at densities appropriate for each assay, as indicated.

The MLS-9 cell line was derived in our laboratory after treating primary rat microglia with colony-stimulating factor-1 for several weeks [24]. Cells were thawed and cultured for several days in medium (MEM, 10% FBS, 100 µM gentamycin), and then harvested in phosphate buffered saline (PBS) containing 0.25% trypsin and 1 mM EDTA, washed with MEM, centrifuged (300×g, 10 min) and re-suspended in culture medium. We routinely use MLS-9 cells to study Cl^−^ channels [25] and several K^+^ channels; i.e., KCa2.3, KCa3.1 [21], [26], Kv1.3 [27] and ERG [24], [28], [29]. There are several advantages of using MLS-9 cells for studying KCa and TRPM7 currents. They lack two interfering K^+^ currents – inward-rectifier (Kir2.1) and Kv1.3 [30], [31] – and they have large KCa2.3 [21] and TRPM7 currents (present study).

### Chemicals

Primary microglia and MLS-9 cells were stimulated with 20 ng/ml rat recombinant IL4 or 20 ng/ml rat recombinant IL10 (both from R&D Systems Inc., Minneapolis, MN). These concentrations were based on previous work from our laboratory [13], [21] and others: IL4 [32], [33] and IL10 [33], [34], [35]. Several channel inhibitors were used to assess contributions of ion channels. Apamin (Sigma-Aldrich, Oakville, ON, Canada) is a well-known pore blocker of KCa2.3 channels (and KCa2.1 and 2.2) at higher doses (IC_50_ = 4 nM) but only KCa2.1 and 2.2 at lower doses (IC_50_ = 704 pM and 27 pM, respectively) [36]. Similarly, tamapin (Alamone Labs, Jerusalem, Israel) is a potent pore blocker of KCa2.3 (and KCa2.2) channels at higher concentrations (IC_50_ = 1.7 nM) but only KCa2.2 at lower concentrations (IC_50_ = 24 pM) [37]. NS8593 (Sigma-Aldrich) was developed as a negative gating modulator (inhibitor) of KCa2.1, KCa2.2 and KCa2.3 channels, and is effective at sub-micromolar levels with physiologically relevant intracellular Ca^2+^ concentrations [38]. However, NS8593 was recently found to block cloned TRPM7 channels [18]. AA-861 (Sigma-Aldrich) is a 5-lipooxygenase inhibitor that was reported to effectively inhibit TRPM7 channels [39]. Stock solutions were made in sterile PBS with 0.3% bovine serum albumin (IL4, IL10), sterile double distilled water (apamin, tamapin) or ethanol (AA-861). For all channel inhibitors, aliquots were stored at −20°C.

### Gene expression assays

#### RNA extraction

Total RNA was extracted using TRIzol reagent (Invitrogen), and an RNeasy Mini Kit (QIAGEN, Mississauga, ON, Canada), as in our recent studies [13], [21], [23]. RNA samples were stored at −80°C.

#### Multiplexed gene expression analysis (NanoString nCounter)

This high-throughput method was used to analyze molecular markers of microglial activation, as well as matrix degrading enzymes and other molecules of particular relevance to this study (e.g., the *KCNN3*/KCa2.3 channel). Each gene (Table 1) was recognized by a probe set (consisting of a capture probe and a reporter probe) that was designed and synthesized by NanoString nCounter technologies. Extracted RNA (200 ng) was sent to the Princess Margaret Genomics Centre (http://www.pmgenomics.ca; Toronto, Canada), where the sample purity was assessed (using Nanodrop 1000) before conducting the assay (hybridization, detection, scanning) according to the manufacturer's instructions. Background subtraction and normalization of RNA counts were conducted using NanoString nCounter digital analyzer software (http://www.nanostring.com/support/ncounter/). Gene expression was normalized to the housekeeping gene, hypoxanthine guanine phosphoribosyl transferase *(HPRT1)*, which we find to be stable in rat primary microglia under all treatments we have investigated [13], [21], [23].

**Table 1:** Target sequences used to design probe sets for NanoString nCounter analysis.

Gene	Genbank Accession #	Target Sequence
Arg1	NM_017134.2	ACGGGAAGGTAATCATAAGCCAGAGACTGACTACCTTAAACCACCGAAATAAATGTGAATACATCGCATAAAAGTCATCTGGGGCATCACAGCAAACCGA
Ctsb	NM_022597.2	GTCTGGGAAAAACCTGCTTTTTTGTTGTAGGTGCCACGTAACCCTGTCAGTTTAACAAGGAATGACCGTGCCAATAAACCAATTCTCCCTCTGCTTGAAA
Ctsd	NM_134334.2	GACACTGTGTCGGTTCCATGTAAGTCAGACTTAGGAGGTATCAAGGTGGAGAAACAGATCTTTGGGGAAGCCACCAAGCAGCCTGGAGTCGTATTCATCG
Ctsk	NM_031560.2	AGCAGGCTGGAGGACTAAGGTGACCTTCCCAAGCCCCTGTCTTCTATATACACCAGTGCAGATTTCAGTCTTCCACTGAGATGCACAAATCTATTCATGA
CtsL1	NM_013156.1	GTGGGGCCTATTTCTGTTGCCATGGATGCAAGCCATCCGTCTCTCCAGTTCTATAGTTCAGGTATCTACTATGAACCCAACTGTAGCAGCAAGGACCTCG
Ctss	NM_017320.1	GATTGCTCAACCGAAGAAAAGTACGGGAATAAAGGCTGCGGGGGTGGCTTCATGACCGAAGCTTTCCAGTACATCATCGATACGAGCATCGACTCAGAAG
Itgam/CD11b	NM_012711.1	CATCCCTTCCTTCAACAGTAAAGAAATATTCAACGTCACCCTCCAGGGCAATCTGCTATTTGACTGGTACATCGAGACTTCTCATGACCACCTCCTGCTT
CD163	NM_001107887.1	AGTTTCCTCAAGAGGAGAGGTCTTGATACATCAAGTTCAGTACCAAGAGATGGATTCGAAGACGGATGATCTGGACTTGCTGAAATCCTCGGGTTGGCAT
c-myc	NM_012603.2	ACCGAGGAAAACGACAAGAGGCGGACACACAACGTCTTGGAACGTCAGAGGAGAAACGAGCTGAAGCGTAGCTTTTTTGCCCTGCGCGACCAGATCCCTG
CD68/ED1	NM_001031638.1	CTCTCATTCCCTTACGGACAGCTTACCTTTGGATTCAAACAGGACCGACATCAGAGCCACAGTACAGTCTACCTTAACTACATGGCAGTGGAATACAATG
Hpse	NM_022605.1	CTTGAATGGACGAGTTGCGACCAAAGAAGATTTTCTGAGCTCTGATGTCCTGGACACTTTTATCCTATCTGTGCAAAAAATTCTGAAGGTGACTAAGGAG
HO-1/Hsp32	NM_012580.2	CTAGTTCATCCCAGACACCGCTCCTGCGATGGGTCCTCACACTCAGTTTCCTGTTGGCGACCGTGGCAGTGGGAATTTATGCCATGTAAATGCAGTGTTG
Il1b	NM_031512.1	TGCACTGCAGGCTTCGAGATGAACAACAAAAATGCCTCGTGCTGTCTGACCCATGTGAGCTGAAAGCTCTCCACCTCAATGGACAGAACATAAGCCAACA
Il1rn/Il1ra	NM_022194.2	TCATTGCTGGGTACTTACAAGGACCAAATACCAAACTAGAAGAAAAGATAGACATGGTGCCTATTGACTTTCGGAATGTGTTCTTGGGCATCCACGGGGG
Il6	NM_012589.1	GGAACAGCTATGAAGTTTCTCTCCGCAAGAGACTTCCAGCCAGTTGCCTTCTTGGGACTGATGTTGTTGACAGCCACTGCCTTCCCTACTTCACAAGTCC
Nos2/iNOS	NM_012611.2	ACGGGACACAGTGTCGCTGGTTTGAAACTTCTCAGCCACCTTGGTGAGGGGACTGGACTTTTAGAGACGCTTCTGAGGTTCCTCAGGCTTGGGTCTTGTT
Mmp2	NM_031054.2	CTTTTTTGTGCCCAAAGAAAGGTGCTGACCGTATCCCTCCCAGGTGCTACTTTCTCCCGCCCACCCAAGGGGATGCTTGGATATTCACAATGCAGCCCTC
Mmp9	NM_031055.1	TGCGTCGGGCGCTGCTCCAACTGCTGTATAAATATTAAGGTATTCAGTTACTCCTACTGGAAGGTATTATGTAACCATTTCTCTCTTACATCGGAGGACA
Mmp12	NM_053963.1	AGGCACAAACCTGTTCCTTGTTGCTGTTCATGAGCTTGGCCATTCCTTGGGGCTGCGGCATTCCAATAATCCAAAATCAATAATGTACCCTACCTACAGA
Mmp14	NM_031056.1	GTTCCAGATAAGTTTGGGACTGAGATCAAGGCCAATGTTCGGAGGAAGCGCTATGCCATTCAGGGCCTCAAGTGGCAGCATAATGAGATCACTTTCTGCA
Mrc1	NM_001106123.1	CTTTGGAATCAAGGGCACAGAGCTATATTTTAACTATGGCAACAGGCAAGAAAAGAATATCAAGCTTTACAAAGGTTCCGGTTTGTGGAGCAGATGGAAG
Nfe2l2/Nrf2	NM_031789.1	ATACAACAAAAAAAGAAGTACCTGTGAGTCCTGGTCATCAAAAAGTCCCATTCACAAAAGACAAACATTCAAGCCGATTAGAGGCTCATCTCACAAGAGA
Stat6	NM_001044250.1	GTGGTTTGATGGTGTCCTGGACCTCACTAAACGCTGTCTTCGGAGCTACTGGTCAGATCGGCTGATCATCGGCTTTATCAGTAAGCAATATGTCACTAGC
Tgfb1	NM_021578.2	CGCCTGCAGAGATTCAAGTCAACTGTGGAGCAACACGTAGAACTCTACCAGAAATATAGCAACAATTCCTGGCGTTACCTTGGTAACCGGCTGCTGACCC
Timp1	NM_053819.1	CATCGAGACCACCTTATACCAGCGTTATGAGATCAAGATGACTAAGATGCTCAAAGGATTCGACGCTGTGGGAAATGCCACAGGTTTCCGGTTCGCCTAC
Tlr2	NM_198769.2	TTTACAAACCCTTAGGGTAGGAAATGTTGACACTTTCAGTGAGATAAGGAGAATAGATTTTGCTGGGCTGACCTCTCTCAACGAACTTGAAATTCAGGTA
Tlr4	NM_019178.1	GTCAGTGTGCTTGTGGTAGCCACTGTAGCATTTCTGATATACCACTTCTATTTTCACCTGATACTTATTGCTGGCTGTAAAAAGTACAGCAGAGGAGAAA
Tnf	NM_012675.2	GGTGATCGGTCCCAACAAGGAGGAGAAGTTCCCAAATGGGCTCCCTCTCATCAGTTCCATGGCCCAGACCCTCACACTCAGATCATCTTCTCAAAACTCG

#### Quantitative real-time reverse-transcriptase polymerase chain reaction (qRT-PCR)

RNA samples were reverse transcribed using SuperScript II reverse transcriptase, according to the manufacturer's instructions (Invitrogen). The following primers for *TRPM7* and the housekeeping gene, *HPRT1*, were designed using ‘Primer3Output’ (http://bioinfo.ut.ee/primer3-0.4.0). *TRPM7*: forward (5′- AGGGCAGTGGTTTGCTGT-3′) and reverse (5′- CAGGGCCAAAAACCATGT-3′). *HPRT1*: forward (5′- CAGTACAGCCCCAAAATGGT-3′) and reverse (5′- CAAGGGCATATCCAACAACA-3′). cDNA was amplified using an ABI PRISM 7700 Sequence Detection System (PE Biosystems, Foster City, CA, USA) as follows: 50°C for 2 min, 95°C for 10 min, 40 cycles at 95°C for 15 sec, and 60°C for 60 sec; followed by a dissociation step (95°C for 15 sec, 60°C for 15 sec, 95°C for 15 sec). ‘No-template’ and ‘no-amplification’ controls were included for both genes, and single peaks on the melting curves confirmed the specific amplification of each gene. The threshold cycle (CT) for *TRPM7* was normalized to that of *HPRT1* before analyzing and comparing gene expression.

### Immunocytochemistry

The methods were similar to our recent papers [13], [16], [17], [21]. Briefly, microglia or MLS-9 cells were seeded at 70,000–80,000 cells per UV-irradiated, 15 mm glass coverslip (Fisher Scientific, Ottawa, ON, Canada), cultured for 1–2 days in MEM with 2% FBS, and then fixed in 4% paraformaldehyde (Electron Microscopy Sciences, Hatfield, PA, USA) at room temperature for 15 min. Cells were permeabilized with 0.2% Triton X-100 for 5 min and washed in PBS (3×, 5 min each). To visualize filamentous (F) actin, cells were incubated with Acti-stain 488 phalloidin (Cytoskeleton Inc., Denver, CO, USA) at 1∶100 in PBS for 1 hr at room temperature. Cell nuclei were labeled with 4′,6-diamidino-2-phenylindole (DAPI; Invitrogen) at 1∶3000 in PBS for 5 min. After washing (3×, 5 min each), cells on coverslips were mounted on glass slides with Dako mounting medium (Dako, Glostrup, Denmark) and stored at 4°C.

### Transmigration and invasion assays

For transmigration assays, microglia were seeded at 40,000 cells/well on the upper well of Transwell filter inserts (VWR, Mississauga, ON, Canada). The filters contain 8 µm-diameter pores that allow cell haptokinesis; i.e., random migration without an applied chemical gradient. For invasion assays, the setup was the same, except the cells were seeded on BioCoat Matrigel Invasion Chambers (BD Biosciences, Mississauga, ON, Canada), in which the filters are coated with Matrigel, a basement membrane-like ECM substance secreted by mouse sarcoma cells. Cells must degrade the Matrigel in order to migrate to underside of the filter. One hour after seeding, MEM with 2% FBS was added to both upper and lower wells, with or without 20 ng/ml IL4 or IL10. After 1 hr further incubation, a channel inhibitor was added (see ‘Chemicals’). The chambers were then incubated for 24 hr (37°C, 5% CO_2_), and the filters were fixed in 4% paraformaldehyde for 10 min and rinsed with PBS. Microglia that remained on the upper side of the filter were removed by swirling a Q-tip on the filter surface. To visualize microglia that had translocated to the underside of the filter, cells were stained with 0.3% crystal violet in methanol solution for about 1 min, and rinsed with PBS to remove excess stain. The cells were counted in 5 random fields/filter at 40× magnification, using an Olympus CK2 inverted microscope (Olympus, Tokyo, Japan).

### Patch-clamp electrophysiology

Primary rat microglia and MLS-9 cells were plated on 15 mm diameter coverslips at ∼7.5×10^4^/coverslip and mounted in a model RC-25 perfusion chamber (Warner Instruments, Hamden, CT). They were superfused with an extracellular (bath) solution containing (in mM): 125 NaCl, 5 KCl, 1 MgCl_2_, 1 CaCl_2_, 5 glucose, and 10 HEPES, adjusted to pH 7.4 (with NaOH). Sucrose was added to adjust the osmolarity to ∼310 mOsm (measured with a freezing point-depression osmometer; Advanced Instruments, Norwood, MA), which prevented activation of the swelling-activated Cl^−^ current (Schlichter et al., 2011). Bath solutions were exchanged using a gravity-driven perfusion system flowing at 1.5–2 ml/min. All recordings were made at room temperature. Recordings were performed in the whole-cell- or perforated-patch configuration using 7–9 MΩ resistance pipettes pulled from thin-walled borosilicate glass (WPI, Sarasota, FL) using a Narishige puller (Narishige Scientific, Setagaya-Ku, Tokyo). Patch-clamp data were obtained with an Axon Multiclamp 700A amplifier (Molecular Devices, Sunnyvale, CA). Signals were compensated on-line for capacitance and series resistance, filtered at 5 kHz, and acquired and digitized using a Digidata 1322A board with pClamp software (ver9; Molecular Devices). Junction potentials were reduced by using agar bridges made with bath solution, and were calculated with the utility in pClamp. After correction, all voltages were about 5 mV more negative than shown.

TRPM7 currents were recorded in the whole-cell configuration with a pipette solution buffered to ∼20 nM free Ca^2+^ to preclude activation of KCa2.3 channels. Because these channels are inhibited by high intracellular Mg^2+^ in microglia [19] and other cells [40], [41], most recordings used a Mg^2+^-free pipette solution containing (in mM): 100 K-aspartate, 40 KCl, 1 CaCl_2_, 2 K_2_ATP, 10 EGTA, 10 HEPES, pH adjusted to 7.2 with KOH, 280 mOsm/kgH_2_O. The exception was for experiments testing the Mg^2+^-dependence of NS8593, in which case free Mg^2+^ was adjusted to 75 or 300 µM by adding 1 mM or 2 mM MgCl_2_, respectively to the standard pipette solution. Free Mg^2+^ (and Ca^2+^, below) concentrations were calculated with WEBMAXC Extended software (http://www.stanford.edu/~cpatton/webmaxc/webmaxcE.htm). KCa2.3 currents were recorded in the perforated-patch configuration, using pipettes containing 200 µg/ml amphotericin B (Sigma), in a similar ionic solution, but with 0.5 CaCl_2_ and 1 EGTA to obtain ∼120 nM free Ca^2+^. After a giga-ohm seal formed, amphotericin B lead to a gradual decrease in series resistance, and experiments were begun after the resistance was <100 MΩ.

### Statistical analysis

All graphical data are presented as mean ± SEM, with the number of cells or samples indicated on each bar. Data were analyzed with a one-way ANOVA followed by Tukey's test or a two-way ANOVA followed Bonferroni's test, using GraphPad Prism ver 5.01 (GraphPad Software, San Diego, CA). Results were considered significant if *p*<0.05.

## Results

### Changes in gene expression indicate similarities and differences in microglial responses to IL4 and IL10

**Figure 1:**
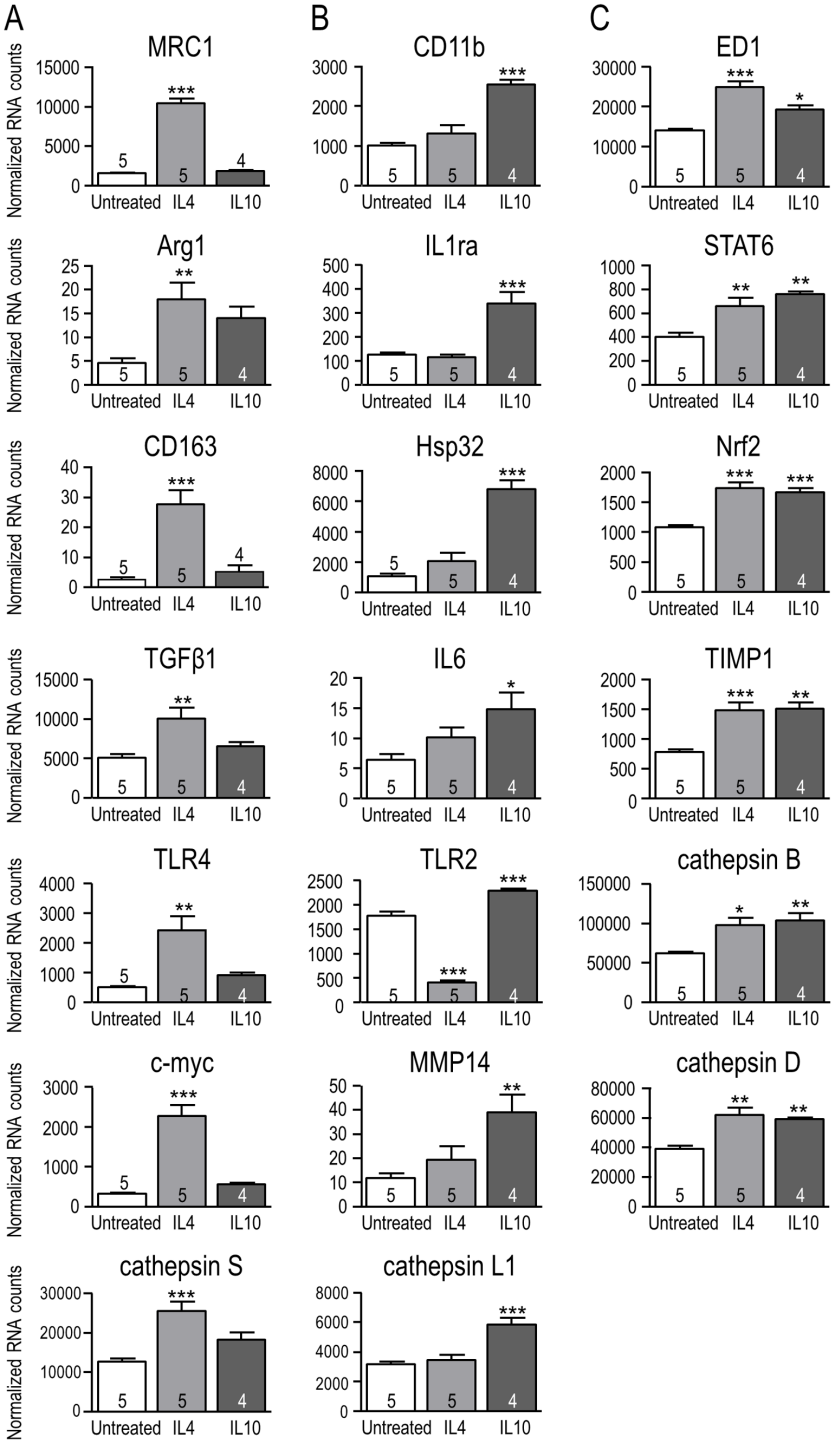
NanoString analysis of expression changes for selected genes in response to IL4- and IL10-treatment. Treating rat primary microglia for 6 hr with 20 ng/ml IL4 or 20 ng/ml IL10 resulted in gene expression changes that were specific for IL4 (**A**), specific for IL10 (**B**), or common to both treatments (**C**). mRNA expression was normalized to the housekeeping gene, *HPRT1*, and shown as mean ± SEM with the number of individual cultures indicated on each bar. [Note the differing Y-axis scales.] One-way ANOVA with Tukey's post-hoc test revealed differences from unstimulated (control) microglia: *p<0.05, **p<0.01, ***p<0.001.

Microglial cells were stimulated for 6 hr with IL4 to induce ‘alternative’ activation or with IL10 to induce ‘acquired deactivation’ (see Introduction). This early time point was chosen to allow time for protein changes to occur and exert effects during the hr functional assays. The multiplex NanoString assay was used to compare mRNA expression of 28 genes (listed in Table 1) within several categories: well-known markers of classical activation (e.g., IL1β, TNFα, iNOS, IL6) and alternative activation (MRC1, Arg1, CD163); other known immune mediators; and matrix-degrading enzymes of relevance to microglial migration and invasion. The rapidly induced genes fell into four groups: those induced selectively by IL4 (Fig. 1A), selectively by IL10 (Fig. 1B), by both cytokines (Fig. 1C), or unaffected by either cytokine (not shown). Note that the 6 hr results do not rule out later changes in gene expression. The findings described below indicate that IL4 and IL10 induce different expression patterns in rat microglia despite both being considered anti-inflammatory cytokines.

***(i) IL4-specific induction.*** IL4 alone increased transcript levels of 7 of the genes examined, including four hallmark molecules of alternative activation: mannose receptor C type 1 (MRC1), arginase 1 (Arg1), CD163, transforming growth factor-β1 (TGFβ1) by 5.5-, 2.9-, 9.8- and 2-fold, respectively. These changes are similar to our recent results [13], [21]. The transcription factor, c-myc, which polarizes human macrophages toward an alternative-activation state [42], was increased 6.2 fold. The toll-like receptors (TLRs) exemplify differential effects of the cytokines on gene expression. IL4 increased TLR4 by 3.8 fold and decreased TLR2 by 77%. Among the matrix-degrading enzymes examined, only IL4 increased cathepsin S. ***(ii) IL10- specific induction.*** IL10 selectively up-regulated 7 of the genes examined. CD11b, a subunit of complement receptor 3 that is often used to identify microglia, was increased 1.5 fold. We examined two genes that help dampen effects of pro-inflammatory stimuli: the IL1 receptor antagonist (IL1ra) and the anti-oxidant, heme oxygenase-1 (HO-1; also called Hsp32) [43], [44]: IL1ra was increased 1.7 fold and Hsp32 by 5.4 fold. IL10 increased expression of the pleiotropic cytokine, IL6, by 1.3 fold. IL10 increased TLR2 by 1.3 fold, but had no effect on TLR4. IL10 up-regulated the matrix-degrading enzymes, cathepsin L1 (1.8 fold) and MMP14 (2.3 fold). ***(iii) Induced by both IL4 and IL10.*** Six of the genes examined were up-regulated by both IL4 and IL10. ED1, which is a lysosomal marker often used to indicate microglial activation and phagocytic capacity, was increased 1.8 fold by IL4 and 1.4-fold by IL10. STAT6 is an important signaling molecule linked to IL4-induced alternative activation (reviewed in [1], [45]): it was increased 1.6 fold by IL4 and 1.9 fold by IL10. The transcription factor, nuclear factor-erythroid 2-related factor 2 (Nrf2), regulates redox homeostasis and skews microglia toward alternative activation [46]: it was increased 1.6 fold by IL4 and 1.5 fold by IL10. The endogenous broad-specificity MMP inhibitor, TIMP metallopeptidase inhibitor 1 (TIMP1), was increased equally (1.9 fold) by IL4 and IL10. IL4 and IL10 both increased the matrix-degrading enzymes, cathepsin B (1.6- and 1.7 fold, respectively) and cathepsin D (1.6- and 1.5 fold, respectively). ***(iv) Not induced (not shown).*** Neither cytokine significantly altered expression of the pro-inflammatory genes, tumor necrosis factor-α (TNF-α) or inducible nitric oxide synthase (iNOS) at the 6 hr time point. Among the matrix-degrading enzymes, neither cytokine altered expression of heparanase, cathepsin K or the matrix metalloproteases, MMP2 and MMP12. Although not reaching statistical significance (n = 5 samples), IL4-treated microglia apparently had less IL1β and more MMP9.

### Podosome expression depends on the microglial activation state but not on KCa2.3 or TRPM7 channels

**Figure 2:**
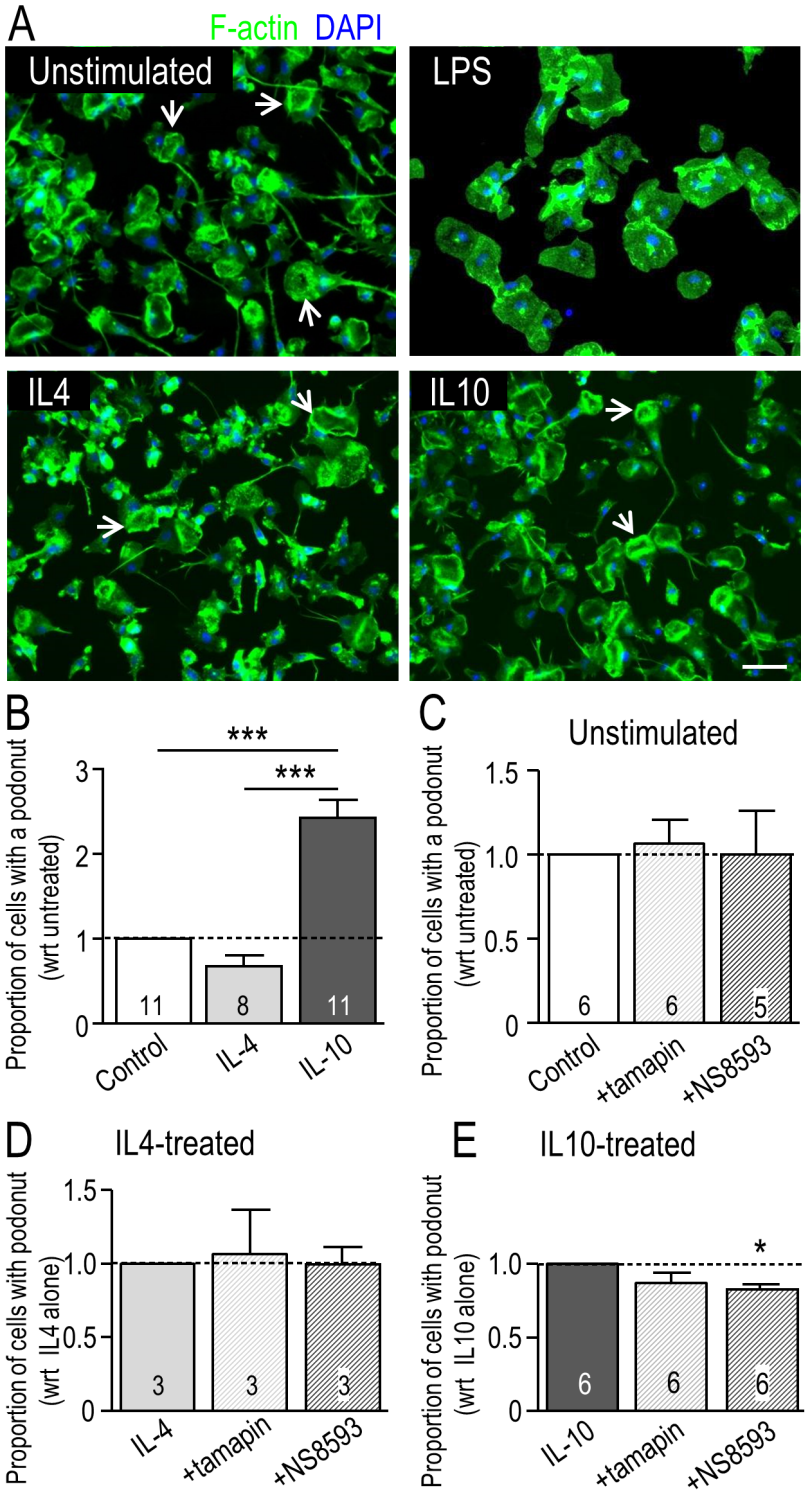
Effect of microglial activation state and selected channel inhibitors on podosome (podonut) expression. **A.** Representative fluorescence micrographs show podonut expression in unstimulated primary rat microglia or 24 hr after treatment with LPS (10 ng/ml), IL4 (20 ng/ml) or IL10 (20 ng/ml). The arrows indicate examples of a single, large donut-shaped ring of podosomes, which we call a ‘podonut’ in the lamellum of unipolar microglia. Cells were stained for filamentous (F-) actin with phalloidin (green) to quantify the proportion of cells with a podonut, and with the nuclear marker, DAPI (blue) to quantify total cell numbers. Scale bar, 50 µm. **B.** Podosome (podonut) expression is affected by the microglial activation state. The proportion of microglial cells bearing a podonut in the lamellum (sum of 3 randomly selected fields of view at 10× magnification) was normalized to unstimulated cells. **C–E.** Microglia were unstimulated or stimulated for 24 hr with 20 ng/ml IL4 or IL10, and then exposed for 6 hr to control medium or the KCa2.3 inhibitors, 5 nM tamapin or 7 µM NS8593, which also inhibits TRPM7 channels (see Results and Figs. 6, 7). All graphical data are shown as mean ± SEM with sample size (# of individual cultures) indicated on each bar. A one-way ANOVA with Tukey's post-hoc analysis was used to determine significant differences. *p<0.05; ***p<0.001.

F-actin is concentrated in the core of each podosome in microglia [16], [17] and other cells (reviewed in [15]), and is thus a useful marker for quantifying the proportion of microglia bearing a podonut. Microglia were treated for 24 hr with LPS, IL4 or IL10 (Fig. 2). At this time, most unstimulated microglia were unipolar with a single, large fan-shaped lamellum at the leading edge and a trailing uropod; i.e., 57% (n = 9 separate cultures) in static counts of fixed cells (Fig. 2A). In contrast, LPS-treated cells were amoeboid or round and flat. Most IL10-treated microglia were unipolar with a lamellum and a uropod (64%; n = 9). The morphology of IL4-treated cells was more variable, but many cells were unipolar with a lamellum that exhibited extensive membrane ruffling (43%; n = 9); consistent with our recent report [13]. These were the morphologies of microglia used for gene-expression analysis in Figure 1. Because LPS-treated microglia did not have podonuts and did not migrate during the 24 hr test period [13], this treatment was not examined further. Podonuts were well expressed in unstimulated, IL4- and IL10-treated microglia (Fig. 2A). The proportion of microglia with a podonut was increased (by 2.4 fold) only in IL10-treated cells (Fig. 2B).

KCa2.3 (SK3) is enriched in the core of podosomes [17]; therefore, we used two KCa2.3 inhibitors to ask whether this channel regulates podosome expression. In order to factor out culture variability, the proportion of microglia with a podonut in IL4- and IL10-treated cells was normalized to its unstimulated counterpart for each culture. Inhibiting KCa2.3 with 5 nM tamapin for 6 hr did not affect podonut expression in unstimulated (Fig. 2C), IL4-treated (Fig. 2D), or IL10-treated microglia (Fig. 2E). The blocker, NS8593 (7 µM, 6 hr), slightly reduced the number of microglia expressing a podonut (by 13%; *p*<0.05) only in IL10-treated microglia (Fig. 2E). Thus, KCa2.3 channel activity is apparently not necessary for podonut expression.

### Migration of primary microglia is increased by IL4 and IL10 and requires TRPM7 (not KCa2.3)

**Figure 3:**
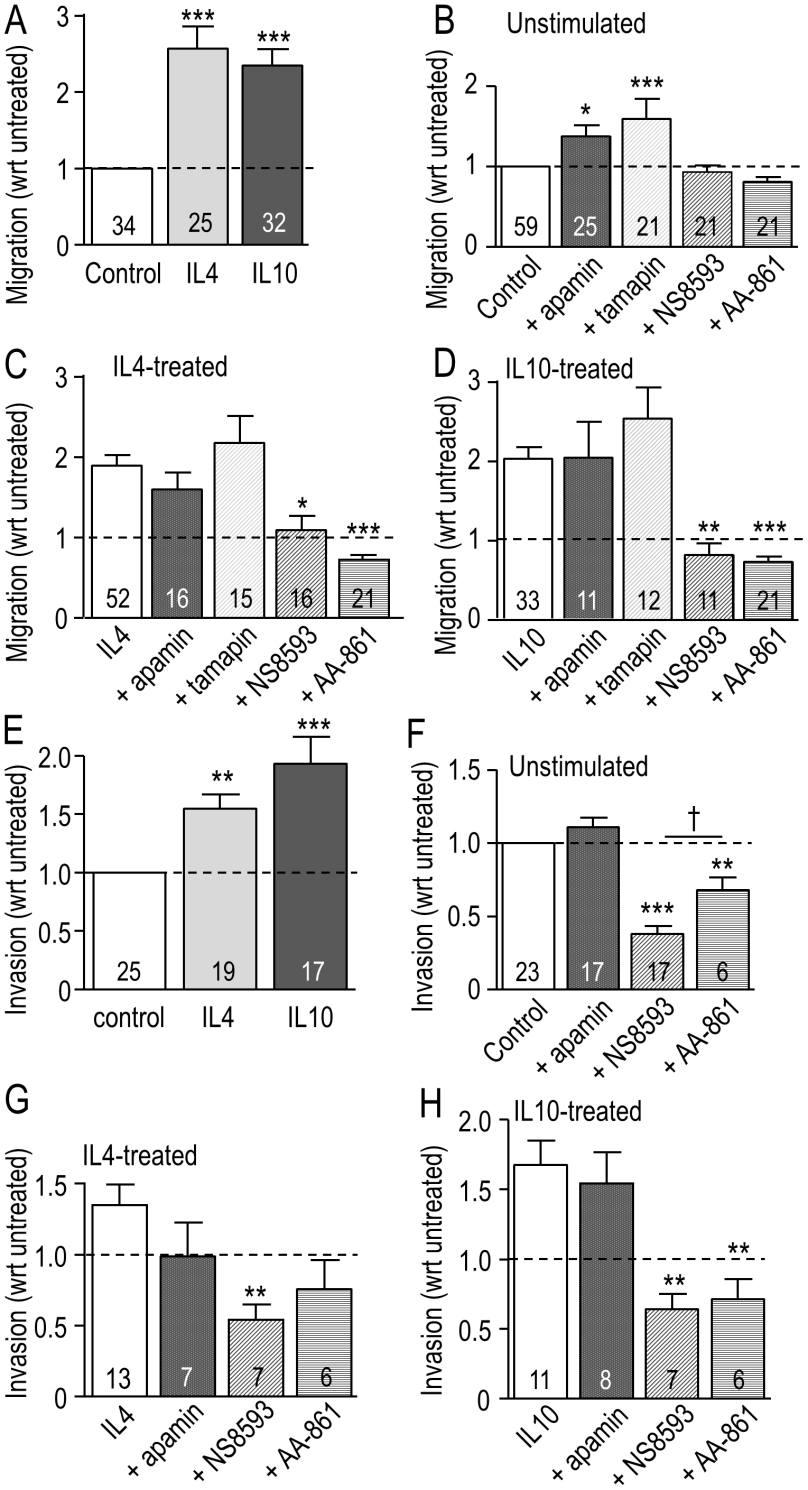
Migration of primary rat microglia is affected by the activation state, and by KCa2.3 and TRPM7 inhibitors. **A.** Microglia in the upper well of Transwells were unstimulated or stimulated with either 20 ng/ml IL4 or 20 ng/ml IL10 for 24 hr. The number of cells that migrated to the underside of the filter was then counted and normalized to control (unstimulated) cells, indicated by the dashed line. **B–D.** Microglia were unstimulated or stimulated with IL4 or IL10 as in panel A, with or without a channel inhibitor: 100 nM apamin, 5 nM tamapin, 7 µM NS8593 or 10 µM AA-861. **E–H.** IL4 and IL10 increase the invasion capacity of rat microglia, and TRPM7 is involved. Microglia were plated in the upper wells of Matrigel chambers, with or without stimulation by 20 ng/ml IL4 or 20 ng/ml IL10 for 24 hr. **E.** Invasion of control (unstimulated) microglia was compared with IL4- and IL10-treated cells. **F–H.** Microglia were unstimulated or stimulated with IL4 or IL10 as in panel A, with or without simultaneous addition of a channel inhibitor: 100 nM apamin, 7 µM NS8593 or 10 µM AA-861. For each stimulus, the number of cells that had migrated or invaded was normalized to the level without a channel inhibitor. The dashed line in all graphs indicates the level in control (unstimulated) cells. Data are expressed as mean ± SEM with the number of individual cultures indicated on each bar. A one-way ANOVA with Tukey's post-hoc test was used to compare results with and without a channel inhibitor; *p<0.05, **p<0.01, ***p<0.001; or for NS8593 versus AA-861 (†p<0.05).

We recently found that microglia stimulated with IL4 migrate much more than unstimulated cells [13]. Because there are some similarities in outcomes and anti-inflammatory effects of IL4 and IL10, we were not surprised that both treatments increased microglia transmigration; by 2.3 fold for IL10 and 2.6 fold for IL4 (Fig. 3A). To assess whether KCa2.3 activity is required for this enhanced migration, we first compared effects of three well known KCa2.3 blockers. Microglia, unstimulated or stimulated with IL4 or IL10, were incubated in Transwell chambers with 5 nM tamapin, 100 nM apamin or 7 µM NS8593. NS8593 was originally described as a selective inhibitor of KCa2.x channels [38]. Based on their known potencies (see Methods), these concentrations should block >95% of KCa2.3 channels for apamin (IC_50_ = 4 nM), 90% for NS8593 (IC_50_ = 0.7 µM), and ∼65% for tamapin (IC_50_ = 1.7 nM). Surprisingly, their effects on transmigration differed. Apamin did not inhibit migration, regardless of the activation state (Fig. 3B–D), and instead, both apamin and tamapin increased migration of unstimulated cells, while NS8593 had no effect (Fig. 3B). [Blocking KCa2.2 alone with 250 pM tamapin had no effect on migration, regardless of cell activation state (data not shown)]. The most striking difference was that NS8593 inhibited transmigration of IL4- and IL10-treated cells (by 50% and 68%, respectively); restoring it to the level of unstimulated microglia. This dramatic reduction by NS8593, but not by apamin or tamapin, indicates that NS8593 is not acting through a block of KCa2.3 channels.

Importantly, NS8593 was recently found to block cloned TRPM7 channels expressed in HEK cells, with an IC_50_ of 1.6 µM in the absence of intracellular Mg^2+^ [18]. This is similar to the IC_50_ for block of KCa2.3, which raises the possibility that the effects of 7 µM NS8593 were due to TRPM7 block. To further address this possibility, we tested AA-861, which inhibits TRPM7 at 10 µM (IC_50_ = 6 µM; [39]). AA-861 preferentially reduced migration of IL4-treated (Fig. 3C) and IL10-treated (Fig. 3D) microglia (by 58% and 65%, respectively), without affecting unstimulated cells (Fig. 3B). These results are entirely consistent with the effects of NS8593 and further indicate that TRPM7, rather than KCa2.3, is involved in the enhanced migration of IL4- and IL10-treated microglia.

### Invasion of primary microglia is increased by IL4 and IL10 treatment and requires TRPM7

When seeded in the upper well of a Matrigel Invasion chamber, microglia must degrade the Matrigel layer in order to invade to the underside of the filter. We previously found that IL4 increased invasion by rat microglia [13] and here, we corroborated that effect and then showed that IL10 increased invasion by 93% compared with unstimulated cells (Fig. 3E). In an earlier study showing that NS8593 inhibited invasion of unstimulated microglia [17], we concluded that KCa2.3 channels were responsible. However, with block of TRPM7 being a possibility, we now compared NS8593 with the KCa2.x blocker, apamin, and the TRPM7 inhibitor, AA-861. Apamin had no effect on invasion regardless of the microglial activation state (Figs. 3E–H). This is consistent with the migration results (Figs. 3A–D), and strongly suggests that despite the presence of KCa2.3 in podosomes, this channel is not needed for substrate (Matrigel) degradation. In contrast, NS8593 reduced invasion of unstimulated, IL4- and IL10-treated cells to a similar degree: ∼60% compared with no channel inhibitor. The TRPM7 inhibitor, AA-861, also decreased microglia invasion: by 57% in IL10-treated cells and 32% in unstimulated cells. Its effect on IL4-treated cells did not reach statistical significance. Overall, it appears that TRMP7 is involved in the invasion capacity of microglia. While for IL4- and IL10-treated cells, reduced invasion might result from their reduced migratory capacity; this is not the full explanation for unstimulated microglia. That is, the TRPM7 blockers reduced invasion but not migration, and thus the channel appears to be involved in substrate degradation.

### Migration of MLS-9 cells is increased by IL4 and IL10, and involves TRPM7 (not KCa2.3)

**Figure 4:**
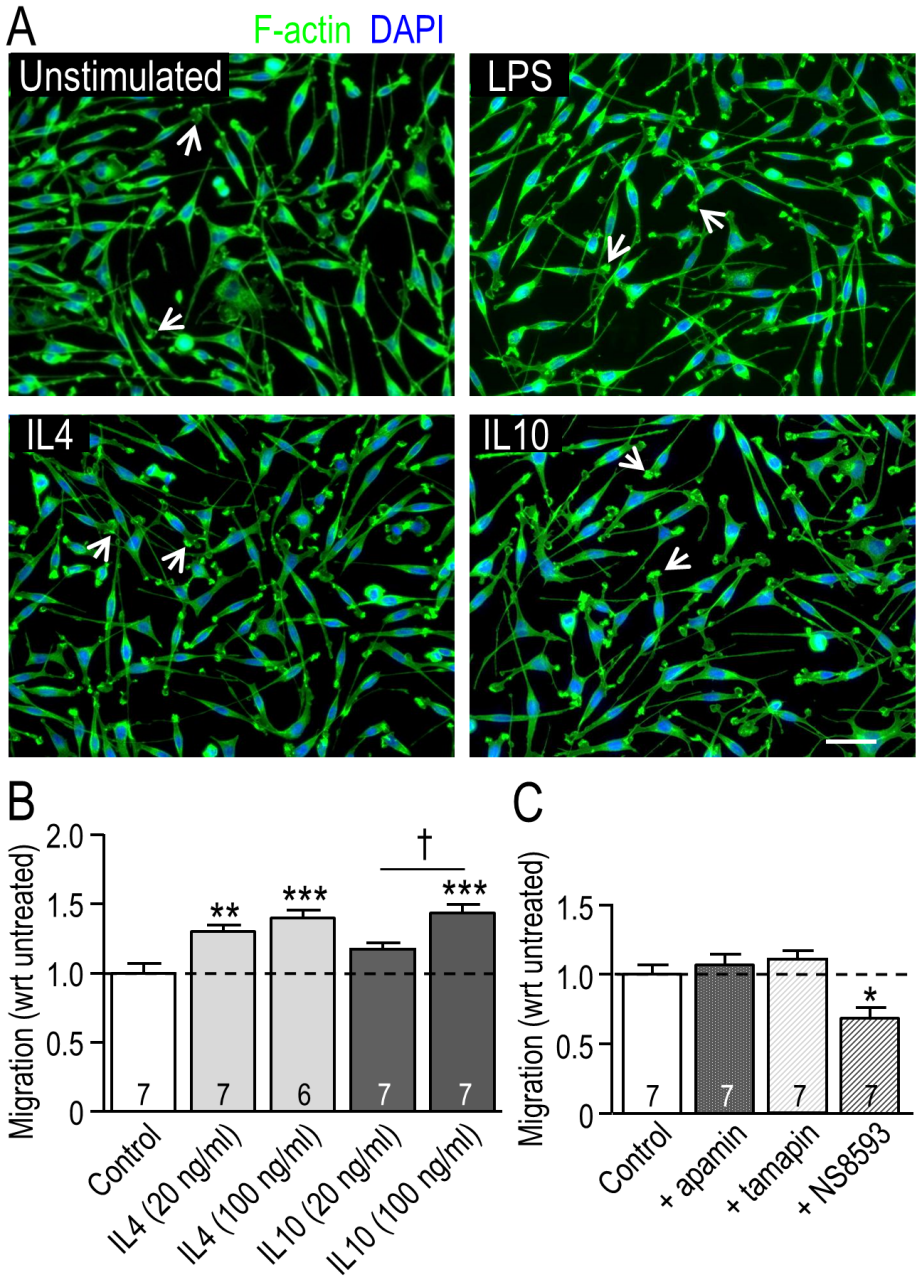
Migration of MLS-9 microglial cells is increased by IL4 and IL10, and involves TRPM7, not KCa2.3. **A.** Representative fluorescence micrographs show MLS-9 cells (rat microglial cell line), unstimulated or 24 hr after stimulation with LPS (100 ng/ml), IL4 (20 ng/ml) or IL10 (20 ng/ml). Cells were stained for F-actin with phalloidin (green) and the nuclear marker, DAPI (blue). Most cells were bipolar with membrane ruffling at one end (examples shown by arrows). Scale bar, 50 µm. **B.** MLS-9 microglial cells in the upper well of Transwells were unstimulated, or stimulated (24 hr) with 20 or 100 ng/ml IL4, or with 20 or 100 ng/ml IL10. The number of cells that migrated to the underside of the filter was then counted and normalized to control (unstimulated) cells. The dashed line in both graphs indicates the level in control (unstimulated) cells. **C.** Cells were stimulated as in panel B, with or without a channel inhibitor: 100 nM apamin, 5 nM tamapin, or 7 µM NS8593. For each stimulus, the number of cells that had migrated was normalized to the level without a channel inhibitor. Data are expressed as mean ± SEM with the number of individual cultures indicated on each bar. In panel B, a two-way ANOVA with Bonferroni post-hoc analysis was used to determine significant differences between stimulation (*) and dose (†). In panel C, A one-way ANOVA with Tukey's post-hoc test was used to determine treatment effects. One symbol, *p*<0.05; two symbols, *p*<0.01; three symbols, *p*<0.001.

The MLS-9 microglia cell line was compared with primary rat microglia because of their utility in studying the KCa2.3 and TRPM7 currents (see below). Unlike primary rat microglia (Fig. 2A), unstimulated MLS-9 cells were mainly bipolar, with membrane ruffling at one end, and LPS, IL4 or IL10 did not alter their morphology (Fig. 4A). MLS-9 cells were migratory and transmigration was increased by IL4 at both concentrations and IL10 at the higher dose (Fig. 4B) but the increase in migration was modest: by 40% with 100 ng/ml IL4 and 43% with 100 ng/ml IL10. The KCa2.3 blockers, apamin and tamapin, did not alter migration but NS8593 reduced migration by 31% (Fig. 4C). Again, this strongly suggests that TRPM7 is involved in MLS-9 cell migration, rather than KCa2.3.

### NS8593 (not AA861) inhibits the KCa2.3 current in microglia

**Figure 5:**
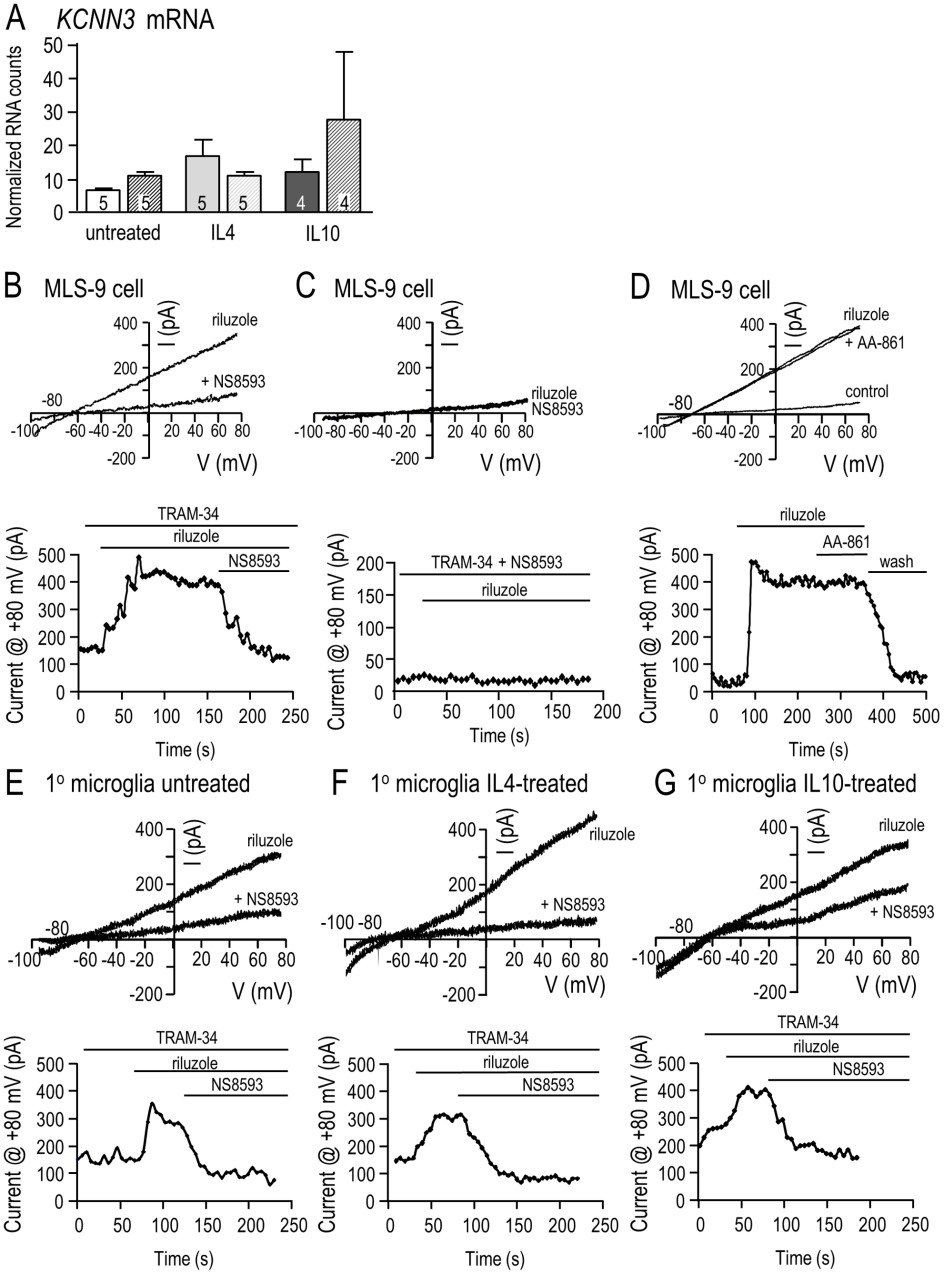
*KCNN3* expression and KCa2.3 current inhibition by NS8593 in microglia in differing activation states. **A.** Expression of *KCNN3* mRNA was quantified using NanoString nCounter analysis in unstimulated primary microglia, and at 6 hr (solid bars) and 24 hr (striped bars) after treatment with 20 ng/ml IL4- or IL10-treatment. **B–D.** NS8593 inhibits the KCa2.3 current, while AA-861 has no effect. KCa2.3 currents were recorded from MLS-9 microglial cells in the perforated-patch configuration produced by amphotericin B (200 µg/ml) in the pipette. The voltage protocol throughout was a 120 ms-long voltage ramp from −100 to +80 mV from a holding potential of −70 mV. The bath always contained 1 µM TRAM-34 to block KCa3.1 currents. Riluzole (300 µM) was used simply as a tool to activate the KCa2.3 current [21]. **B.** *Upper panel*: Representative traces show the current evoked by riluzole with or without 7 µM NS8593. *Lower panel*: The representative time course (current measured at +80 mV) illustrates KCa2.3 current activation and its inhibition by NS8593. **C.** Representative currents and time course show that no current was activated when 7 µM NS8593 was present in the bath. **D.** The current is insensitive to 10 µM AA-861. Note that current activation by riluzole is readily reversible (wash), as we previously showed [21]. **E–G.** The KCa2.3 current in primary rat microglial cells is inhibited by 7 µM NS8593 under differing activation states. Currents were recorded using the same patch-clamp configuration as described for MLS-9 cells. *Upper panels*: Representative currents in microglia that were unstimulated (E), or treated for 24 hr with 20 ng/mL IL4 (F) or 20 ng/mL IL10 (G). *Lower panels*: A representative time course for each cell (current at +80 mV) shows activation by riluzole and inhibition by NS8593.

As shown above, effects of NS8593 on migration of primary microglia during a 24 hr test period depend on the cell activation state (Fig. 3). Results in Figure 5A show a lack of correlation between activation state and expression of *KCNN3*, the gene encoding KCa2.3. There were no significant differences between control (unstimulated), IL4- or IL10-treated microglia at either 6 or 24 hr. Because 7 µM NS8593 had different effects from the KCa2.3 blockers, apamin and tamapin, it was necessary to show whether this drug concentration inhibits KCa2.3 channels in microglia under all three activation conditions. NS8593 was originally described as a negative gating modulator that depends on the intracellular Ca^2+^ concentration; i.e., with a K_d_ of 726 nM at 0.5 µM Ca^2+^ versus ∼14 µM at 10 µM Ca^2+^ [38]. To correspond with NS8593 effects on migration of intact microglia, KCa2.3 inhibition was examined using the perforated-patch configuration to maintain normal intracellular Ca^2+^. Current inhibition was assessed in both MLS-9 microglial cells and primary microglia.

We used riluzole (2-amino-6-(trifluoromethoxy) benzothiazole) simply as a tool to activate KCa2.3 currents because we recently found that it reliably activates KCa2.3 (and KCa3.1) channels in MLS-9 and primary rat microglial cells [21], [22]. To eliminate potential contributions of KCa3.1 currents, the selective KCa3.1 blocker, 1 µM TRAM-34, was added to the bath. ***MLS-9 cells.*** As expected, riluzole activated a KCa2.3 current in all MLS-9 cells tested (15/15; see example in Fig. 5B). The mean current amplitude at +80 mV was 130±15 pA (n = 15). With voltage-clamp ramps from −100 to +80 mV, the current was present at all voltages and reversed near the K^+^ Nernst potential (about −85 mV), as previously described for whole-cell recordings from MLS-9 cells [21]. After the KCa2.3 current was activated, bath perfusion of 7 µM NS8593 rapidly and dramatically inhibited the current in 5/5 cells tested (example in Fig. 5B). With pre-addition of 7 µM NS8593 to the bath (and TRAM-34 to block KCa3.1), no current was activated (6 cells tested; example in Fig. 5C). The observed efficacy of NS8593 is consistent with the reported K_d_ of 726 nM at 0.5 µM intracellular free Ca^2+^ [38]. For completeness, we also tested the TRPM7 inhibitor, 10 µM AA-861, which was used in the migration and invasion assays in Figure 3. AA-861 had no effect on the current (4/4 cells; example shown in Fig. 5D). ***Primary microglia.*** We could find no recordings of KCa2.3 currents in primary rat microglia in the literature. Here, riluzole activated a KCa2.3 current in 1/8 unstimulated microglia (264 pA; Fig. 5D), 2/8 IL4-treated microglia (529 pA, 249 pA; smaller current shown in Fig. 5E), and 2/8 IL10-treated microglia (246 pA, 81 pA; larger current shown in Fig. 5F). The current was very similar to the KCa2.3 current in MLS-9 cells (above). It was present at all voltages tested (−100 to +80 mV) and reversed near the K^+^ Nernst potential, and was consistent with biophysical features of cloned KCa2.3 channels [47]. Due to the low prevalence of current, we did not assess amplitudes in different activation states. In 5/5 microglia, 7 µM NS8593 dramatically inhibited the KCa2.3 current, and this occurred at the normal intracellular free Ca^2+^ concentration maintained by perforated-patch recordings. Pre-treatment with NS8593 could not be tested because KCa2.3 current activation was unreliable in primary microglia.

### NS8593 and AA-861 inhibit the TRPM7 current in microglia

**Figure 6:**
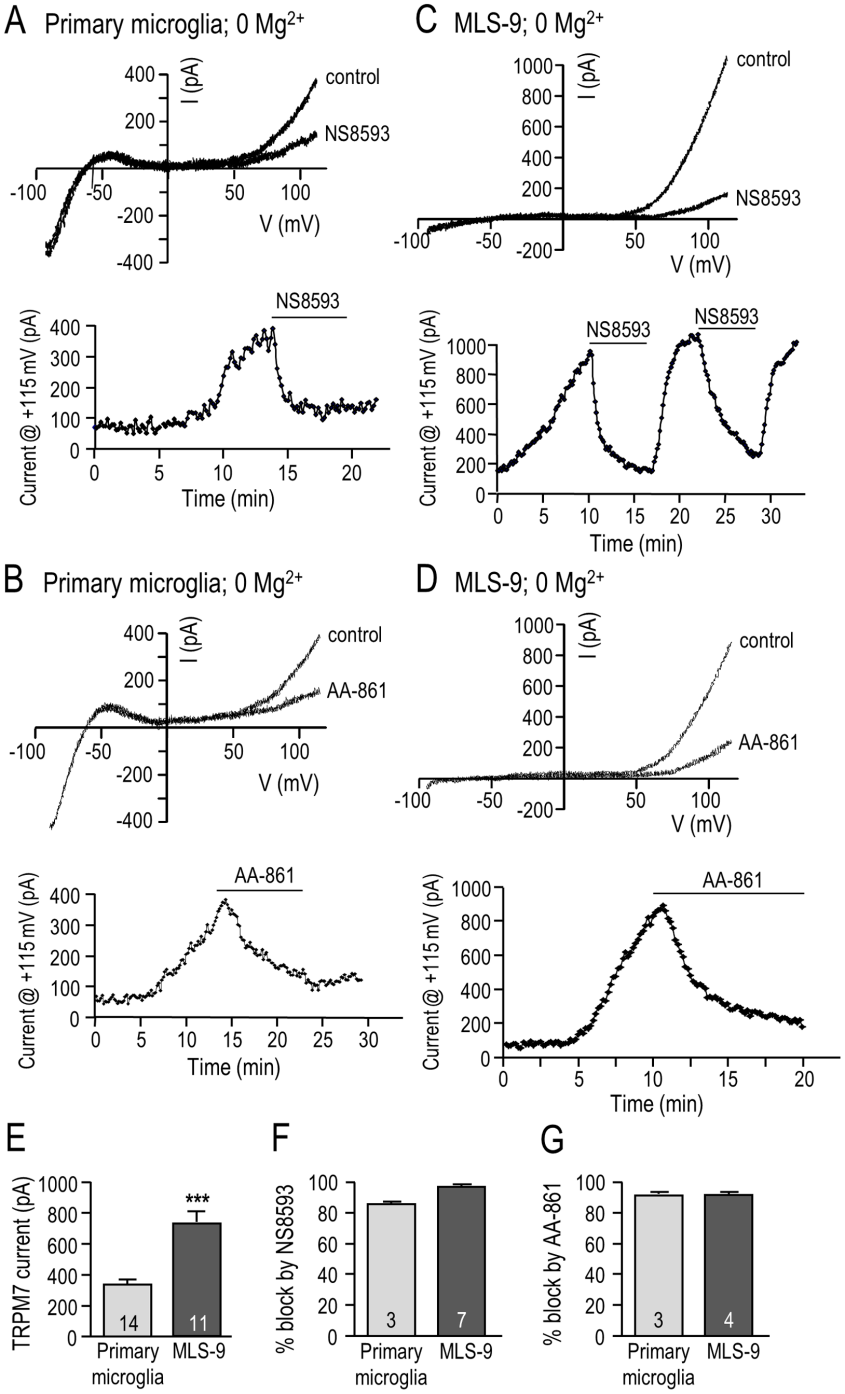
NS8593 and AA-861 inhibit the TRPM7 current in primary rat microglia and MLS-9 microglial cells. TRPM7 currents were recorded in the whole-cell configuration using a Mg^2+^-free pipette solution. The voltage protocol throughout was a 120 ms-long ramp from −100 to +115 mV, from a holding potential of −10 mV. Graphical data are presented as mean ± SEM for the number of cells indicated on each bar, and were compared using an unpaired t-test: ***p<0.001. **A–D.** Representative currents from primary rat microglial cells (A, B) and MLS-9 microglial cells (C, D) with Mg^2+^-free intracellular solution (no MgCl_2_, 10 mM EGTA). *Upper panels*: Currents are shown with and without 7 µM NS8593 (A, C) or 10 µM AA-861 (B, D) in the bath. *Lower panels*: Representative time courses (current measured at +115 mV) show current activation, and inhibition by 7 µM NS8593 (A, C) or 10 µM AA-861 (B, D). [The reversibility of NS8593 is shown in panel C.] **E.** Comparison of the TRPM7 current amplitude (measured at +115 mV) in primary microglia and MLS-9 cells. **F, G.** Comparison of TRPM7 current inhibition by 7 µM NS8593 (F) and by 10 µM AA-861 (G) in primary microglia and MLS-9 microglial cells.

Because we used 7 µM NS8593 and 10 µM AA-861 in functional studies (Figs. 2–4), we tested whether these drug concentrations inhibit the TRPM7 current in microglia (Fig. 6). First, TRPM7 current was assessed in primary rat microglia using the whole-cell configuration with a Mg^2+^-free intracellular solution because block by NS8593 was reported to be mildly Mg^2+^-dependent [18]. Moreover, we previously found that the TRPM7 current in rat microglia spontaneously activates in Mg^2+^-free conditions [19]. A holding potential of −10 mV was used to inactivate Kv1.3 current [30]. [For consistency, the same protocol was used for MLS-9 cells (below), although they lack Kv1.3 current.] As in our earlier study [19], the TRPM7 current was isolated at positive potentials and was strongly outward-rectifying, while an inward-rectifying (Kir2.1) current was present at very negative potentials (Fig. 6A, B). In primary microglia, the TRPM7 current was reduced by both NS8593 and AA-861, while the inward rectifier was not affected. In the examples shown, the time course of the TRPM7 current shows slow, spontaneous activation after break-in, which required more than 10 min to reach a quasi-stable plateau. TRPM7 inhibition by 7 µM NS8593 was rapid (Fig. 6A), while 10 µM AA-861 was slower (Fig. 6B). Similar results were seen with MLS-9 microglial cells (Fig. 6 C, D), which were advantageous in lacking Kv1.3 and Kir2.1 currents, and having much larger TRPM7 currents (721±63 pA *versus* 334±26 pA in primary microglia; Fig. 6E) that activated more rapidly after break-in. Inhibition by NS8593 was readily reversible, which is consistent with direct channel block [18]. [Reversibility in primary microglia was difficult to study because the TRPM7 current activated slowly.] Interestingly, there was a similar degree of inhibition of TRPM7 by both drugs, and in both cell types. NS8593 reduced the current by 86% and 93% in primary microglia and MLS-9 cells, respectively (Fig. 6F). AA-861 reduced the current by 94% and 90% in primary microglia and MLS-9 cells, respectively (Fig. 6G).

### NS8593 inhibits TRPM7 with or without intracellular Mg^2+^, and regardless of the microglial activation state

**Figure 7:**
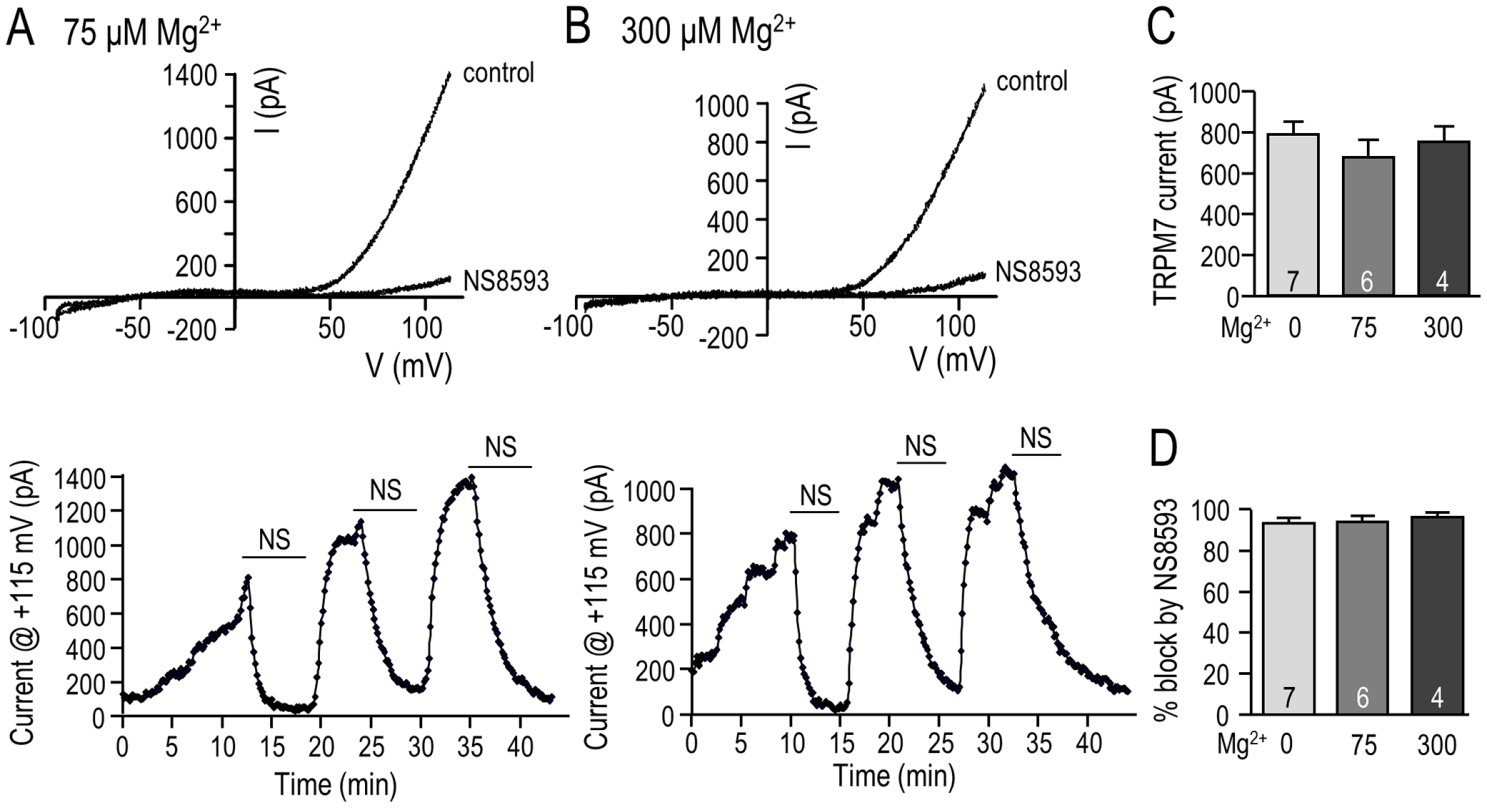
Mg^2+^-dependence of TRPM7 current block by NS8593. TRPM7 currents were recorded in MLS-9 microglial cells in the whole-cell configuration with differing free Mg^2+^ concentrations in the pipette solution. The voltage protocol throughout was a 120 ms-long ramp from −100 to +115 mV, from a holding potential of −10 mV. Graphical data are presented as mean ± SEM for the number of cells indicated on each bar, and were compared using a one-way ANOVA. **A–D.** Effect of 75 µM and 300 µM intracellular free Mg^2+^ on TRPM7 currents and their inhibition by NS8593. In panels A and B, upper traces are representative currents with and without 7 µM NS8593, and the lower panels (time course of current at +115 mV) show the rapid onset and reversibility of inhibition by NS8593. TRPM7 current amplitude (at +115 mV; panel C), and percent inhibition by 7 µM NS8593 (panel D) are compared for 75 µM and 300 µM intracellular free Mg^2+^.

The previously reported TRPM7 block by NS8593 exhibited a mild dependence on intracellular Mg^2+^ (IC_50_ = 1.6 µM without Mg^2+^; 5.9 µM at 300 µM Mg^2+^) [18]. In addition, high intracellular Mg^2+^ inhibits the TRPM7 current in microglia [19] and other cells [48], [49]. Our earlier work [19] showed that the TRPM7 current in primary rat microglia is dose-dependently inhibited by internal Mg^2+^; i.e., the current was reduced ∼25% with 75 µM internal Mg^2+^ and its time-dependent run-up was prevented by 2.8 mM Mg^2+^, a concentration that fully inhibited cloned TRPM7 channels [41]. Normal free Mg^2+^ is 250–1000 µM in mammalian cells [50], and 300 µM Mg^2+^ was reported to reduce the efficacy of TRPM7 inhibition by NS8593 in HEK 293 cells [18]. Therefore, we compared the current amplitude and NS8593 block in MLS-9 cells with 0, 75 and 300 µM intracellular Mg^2+^ (Fig. 6B, Fig. 7). As with Mg^2+^-free intracellular solution (Fig. 6C), the TRPM7 current activated soon after break-in with 75 or 300 µM internal Mg^2+^ (Fig. 7A, B) and reached a quasi-stable plateau by 10 min, at which time the amplitude was measured at +115 mV. Although there might have been a trend toward smaller currents, these levels of Mg^2+^ did not significantly affect the mean amplitude (Fig. 7C): 819±66 pA (no Mg^2+^), 704±87 pA (75 µM Mg^2+^; 15% reduction), and 779±80 pA (300 µM Mg^2+^; 5% reduction). In subsequent experiments, 7 µM NS8593 was added >10 min after break-in. The TRPM7 current was substantially and equally blocked at all three Mg^2+^ concentrations (Fig. 7D): by 93.4% (no Mg^2+^), 93.6% (75 µM Mg^2+^), and 96.2% (300 µM Mg^2+^).

**Figure 8:**
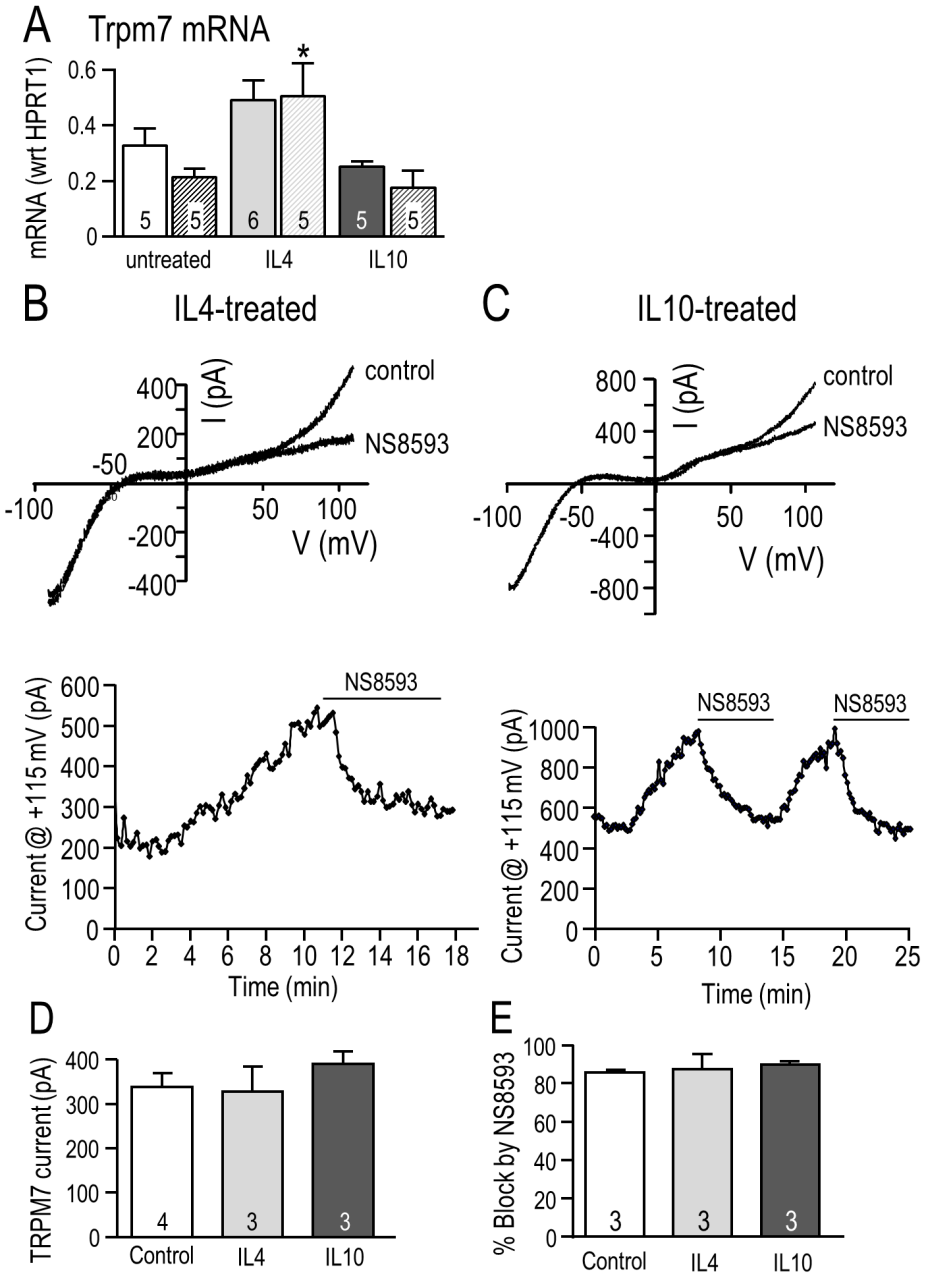
Effects of IL4- and IL10-treatment on TRPM7 expression, current and block by NS8593 in primary rat microglia. **A.** TRPM7 mRNA expression was measured using quantitative real-time RT-PCR at 6 hr (solid bars) and 24 hr (striped bars) in unstimulated rat microglia or after treatment with 20 ng/ml IL4 or 20 ng/ml IL10. Values are expressed as mean expression (normalized to HPRT1) ± SEM, with the number of individual cultures indicated on each bar. *p<0.05 indicates the difference from time-matched control (unstimulated) cells, and was determined using a 2-way ANOVA followed by Bonferroni post-hoc test. **B–E.** The TRPM7 current and block by NS8593 were not affected by 24 hr treatment with 20 ng/ml IL4 or IL10. Whole-cell recordings were performed on primary rat microglia using Mg^2+^-free pipette solution. **B, C.** Representative currents (upper panels) in response to 120 ms-long ramps from −100 to +115 mV, from a holding potential of −10 mV. Time course of the current (lower panels) measured at +115 mV. **D, E.** Summary of TRPM7 current amplitudes measured at +115 mV (**D**) and percent inhibition of TRPM7 currents by 7 µM NS8593 (**E**). Graphical data are presented as mean ± SEM for the number of cells indicated on each bar, and were compared using a one-way ANOVA with Tukey's post-hoc test.

Finally, because NS8593 abolished the enhanced migratory capacity of IL4- and IL10-treated primary microglia, but not unstimulated cells (Fig. 3), we compared TRPM7 expression, current amplitude and block by NS8593 under the three activation conditions (Fig. 8). Treating primary microglia with IL4 (but not IL10) modestly increased TRPM7 expression: at 24 hr it was 1.4-fold higher (Fig. 8A). The TRPM7 current (Fig. 8B, C) and its amplitude (measured at +115 mV; Fig. 8D) were similar under all three conditions; i.e., 339±31 pA in unstimulated microglia, 328±56 pA after IL4, and 390±28 pA after IL10. Moreover, the degree of TRPM7 block by 7 µM NS8593 was the same (Fig. 8E): 85.6% in unstimulated, 87.6% after IL4, and 89.8% after IL10. In summary, 7 µM NS8593 effectively blocks TRPM7 channels in rat microglia and the MLS-9 microglial cell line at physiologically relevant intracellular Mg^2+^ concentrations, and microglia that were unstimulated or treated with IL4 or IL10.

## Discussion

Microglia express receptors for IL4 and IL10 [5], and stimulation with either cytokine is thought to dampen acute CNS inflammation, facilitate removal of debris, and aid in brain repair by up-regulating anti-inflammatory mediators and neurotrophic factors (reviewed in [1], [6]). Studies have examined IL4-mediated alternative activation and IL10-mediated acquired deactivation *in vitro*, often after pre-treating microglia with a classical-activating stimulus such as LPS [51], [52], [53]. Very few studies have directly compared IL4 and IL10 treatment, and the most extensive gene expression comparison suggests that IL10 outcomes resemble LPS in murine microglia [12]. Moreover, there is evidence that IL10 can be elevated very early in models of ischemia and intracerebral hemorrhage [54], [55] and that microglia can initially undergo alternative activation and then progress to classical activation *in vivo* [56].

In comparing gene induction by IL4 and IL10 treatment of isolated rat microglia, some differences are expected because their signaling pathways diverge. Both cytokines can signal through Jak1 kinase, but key downstream transcription factors are STAT6 for IL4, and STAT3 for IL10 (reviewed in [1], [57]). IL4-induced changes in gene expression have been better characterized than responses to IL10. IL4 up-regulates hallmark molecules of alternative activation (e.g., Arg1, MRC1) in murine microglia (reviewed in [1]), and rat microglia [13], [21]. Here, up-regulated genes were compared at 6 hr after IL4- or IL10-treatment in rat microglia that were mainly migratory and unipolar with a lamellum and a uropod. Some genes were specific to each cytokine treatment and some were shared. IL4 treatment selectively increased MRC1, Arg1, CD163, TGFβ1, c-myc, TLR4, and decreased TLR2. IL10 treatment selectively increased CD11b, IL6, IL1ra, TLR2, and Hsp32 (heme oxygenase-1) at 6 hr, but not IL1β or iNOS. Both cytokines up-regulated expression of ED1, TIMP1, and the transcription factors, STAT6 and Nrf2, which potentially contribute to the gene changes they evoked in common. Despite IL4 and IL10 being considered anti-inflammatory cytokines, evidence that they can evoke different gene expression profiles includes some IL10 responses that are similar to pro-inflammatory stimuli (e.g., LPS) but on a reduced scale. IL10 up-regulated IL1β and iNOS by 6 hr, and by 12 hr IL6 was also increased in murine microglia [10], [12].

Microglia are migratory in the developing CNS, and while migration is limited in the healthy adult, it is prevalent after acute CNS injury (reviewed in [2], [5]. Having observed extensive microglial relocation to the injury site after intracerebral hemorrhage [58], [59], ischemia [60], optic nerve damage [61], [62], and spinal cord injury [63], we realized that invasion through brain tissue will require ECM degradation. Thus, it is notable that microglial migration and invasion were increased by both IL4 and IL10 treatment, while migration of LPS-treated cells was decreased [13]; present study). In addition, differences were seen in expression of ECM-degrading enzymes depending on the microglial activation state. IL4 selectively increased cathepsin S, IL10 increased MMP14 and cathepsin L1, while both cytokines increased cathepsins B and D. The IL10 response was similar to the earlier study using LPS, in which MMP14 and cathepsin L1 were increased [13]. While IL10 increased migration of neutrophils [64] and dendritic cells [65] invasion was not examined in those studies.

Podosomes are thought to be important for migration, invasion and ECM degradation [14], [15], and we previously found that reducing podosome numbers reduced migration and invasion of unstimulated rat microglia [17]. Therefore, we hypothesized that the differing migration and invasion capacities of microglia and their dependence on ion channels would correspond with podosome expression. Consistent with the hypothesis, LPS-treated microglia were not migratory and had few if any podosomes; and conversely, blocking KCa2.3 did not reduce podosome expression, migration or invasion in the other activation states. In contrast, IL10 increased podosome prevalence but IL4 did not; and while migration and invasion of IL4 and IL10-treated cells was substantially inhibited by blocking TRPM7 channels, there was little to no effect on podosome expression. This is consistent with our earlier study showing that the TRPM7 inhibitor, spermine, did not affect podosome expression [17]. One possibility is that the substantial basal level of podosomes in unstimulated microglia is sufficient to support migration and the ECM-degradation needed for invasion.

The KCa2.3 channel is present in lamellipodia and filopodia of neural progenitor cells [66], facilitates migration of some cancer cells [67], [68], [69], and is being considered as a therapeutic target for cancer [70]. In breast cancer cells, KCa2.3 can be activated by Ca^2+^ influx through Ca^2+^-release-activated Ca^2+^ (CRAC) channels, which increases migration [71]. Having discovered that microglial podosomes are enriched in KCa2.3 and Orai1 (the pore-forming subunit of CRAC), and that inhibiting Ca^2+^ entry reduces podosomes and migration [17]; we expected that blocking KCa2.3 channels would inhibit their migration and invasion. Instead, migration of unstimulated rat microglia was moderately increased by the blockers, apamin and tamapin, suggesting that KCa2.3 activity is inhibitory; while these blockers had no effect on IL4- or IL10-treated cells. Invasion was not affected by blocking KCa2.3 (with apamin) under any of the three activation conditions. NS8593 reliably inhibited KCa2.3 current in microglia and MLS-9 cells, as previously reported for cloned KCa2.3 channels [38]. In contrast to apamin and tamapin, NS8593 reduced microglial migration after IL4- or IL10-treatment and invasion under all three activation conditions. A KCa2.3 current was detected in fewer than 25% of primary microglia; thus, we also tested migration of MLS-9 microglial cells, which robustly expressed KCa2.3 currents ([21]; present study). KCa2.3 block by apamin or tamapin did not affect MLS-9 cell migration but NS8593 did.

The discrepancy between effects of NS8593 with those of apamin or tamapin prompted us to investigate the role of TRPM7 channels, which were recently found to be inhibited by NS8593 [18]. NS8593 very effectively inhibited the TRPM7 current in microglia, and functional studies indicate that the channel is important for microglia in anti-inflammatory states. That is, both NS8593 and another TRPM7 inhibitor, AA-861 [39], reduced migration of IL4- and IL10-treated microglia but not unstimulated microglia. As expected, AA-861 greatly inhibited the TRPM7 current in microglia but did not affect KCa2.3. Both TRPM7 inhibitors reduced microglial invasion under all three activation conditions. TRPM7 is a Ca^2+^-permeable channel that is ubiquitously expressed and implicated in several homeostatic cellular functions, including divalent cation regulation, cell survival and proliferation (reviewed in [49], [72], [73]). In the CNS, TRPM7 was first discovered in microglia [19], and more recently has been proposed as a therapeutic target to reduce neurodegeneration after stroke [74]. There is evidence that TRPM7 is involved in migration, especially in cancer cells (reviewed in [75]) and highly migratory fibroblasts [76]. TRPM7 can respond to membrane stretch and mediate localized Ca^2+^ entry at the leading edge of migrating fibroblasts [76] and neuroblastoma cells [77]. It can interact with cytoskeletal components [78], [79] to modulate actomyosin contractility that is required for cell migration [80]. Over-expressing TRPM7 in a neuroblastoma cell line facilitated cell adhesion and formation of podosomes [78] and invadosomes [77], and inhibiting TRPM7 reduced migration of activated T cells [81]. TRPM7 whole-cell currents were of similar amplitude in unstimulated microglia and after treatment with LPS or a phorbol ester [19], as well as IL4 or IL10 (present study); however, the channel is regulated by several mechanisms that might affect the current in intact cells. These include intracellular Mg^2+^ [18], [82], [83], Src tyrosine kinase [19], protein kinase A [84], phosphatidylinositol 4,5-biphosphate (PIP_2_) [85], and others (reviewed in [86]). It might be that TRPM7 is preferentially activated in intact microglia to increase their migration and invasion capacity after IL4 or IL10. For instance, IL4 signaling can activate phosphatidylinositide 3-kinase and the Src-family tyrosine kinase, Fes [87].

NS8593, and several other commonly used activators and inhibitors of KCa2.1–2.3 channels are potent inhibitors of TRPM7 channels [18]. The overlapping pharmacological profiles emphasize the need to consider off-target effects on TRPM7 when using these compounds in functional studies. The microglial activation state appears to strongly influence migration and invasion. LPS-treated, classical-activated rat microglia migrated very poorly, while IL-4-treated (alternative-activated) cells migrated and invaded much better than unstimulated microglia, as did microglia in an acquired deactivation state induced by IL10. If highly migratory microglial cells are maintained in anti-inflammatory states, this might reduce bystander damage while they migrate in the developing CNS and to injury sites after brain damage or disease. The present results also suggest that the selective contribution of TRPM7 to migration/invasion of microglia in the anti-inflammatory state should be considered in the current effort to develop TRPM7 inhibitors for CNS disorders.
